# The impact of COVID-19 on the experiences of patients and their family caregivers with medical assistance in dying in hospital

**DOI:** 10.1186/s12904-023-01191-8

**Published:** 2023-06-13

**Authors:** Eryn Tong, Rinat Nissim, Debbie Selby, Sally Bean, Elie Isenberg-Grzeda, Tharshika Thangarasa, Gary Rodin, Madeline Li, Sarah Hales

**Affiliations:** 1 Department of Supportive Care, Princess Margaret Cancer Centre, University Health Network, 620 University Avenue, Toronto, ON Canada; 2 Department of Psychiatry, Faculty of Medicine, University of Toronto, Toronto, ON Canada; 3 Sunnybrook Health Sciences Centre, Toronto, ON Canada

**Keywords:** Medical assistance in dying, Qualitative research, Assisted dying, COVID-19 pandemic, Canada

## Abstract

**Background:**

The COVID-19 pandemic and its containment measures have drastically impacted end-of-life and grief experiences globally, including those related to medical assistance in dying (MAiD). No known qualitative studies to date have examined the MAiD experience during the pandemic. This qualitative study aimed to understand how the pandemic impacted the MAiD experience in hospital of persons requesting MAiD (patients) and their loved ones (caregivers) in Canada.

**Methods:**

Semi-structured interviews were conducted with patients who requested MAiD and their caregivers between April 2020 and May 2021. Participants were recruited during the first year of the pandemic from the University Health Network and Sunnybrook Health Sciences Centre in Toronto, Canada. Patients and caregivers were interviewed about their experience following the MAiD request. Six months following patient death, bereaved caregivers were interviewed to explore their bereavement experience. Interviews were audio-recorded, transcribed verbatim, and de-identified. Transcripts were analyzed using reflexive thematic analysis.

**Results:**

Interviews were conducted with 7 patients (mean [SD] age, 73 [12] years; 5 [63%] women) and 23 caregivers (mean [SD] age, 59 [11] years; 14 [61%] women). Fourteen caregivers were interviewed at the time of MAiD request and 13 bereaved caregivers were interviewed post-MAiD. Four themes were generated with respect to the impact of COVID-19 and its containment measures on the MAiD experience in hospital: (1) accelerating the MAiD decision; (2) compromising family understanding and coping; (3) disrupting MAiD delivery; and (4) appreciating rule flexibility.

**Conclusions:**

Findings highlight the tension between respecting pandemic restrictions and prioritizing control over the dying circumstances central to MAiD, and the resulting impact on patient and family suffering. There is a need for healthcare institutions to recognize the relational dimensions of the MAiD experience, particularly in the isolating context of the pandemic. Findings may inform strategies to better support those requesting MAiD and their families during the pandemic and beyond.

**Supplementary Information:**

The online version contains supplementary material available at 10.1186/s12904-023-01191-8.

## Background

As one of the most acute public health crises in the past century, the coronavirus disease 2019 (COVID-19) pandemic and related public health measures worldwide have drastically altered dying and grieving experiences [1–4]. Containment and mitigation strategies have led to severe restrictions in hospital visitations and family interactions at the end of life [5]. These restrictions have hindered provision of informal caregiver support and family-centered care, adversely affecting patient wellbeing and quality end-of-life care [6, 7]. There have also been limited opportunities for end-of-life discussions, communication with the healthcare team, and farewell rituals [8–10]. Healthcare providers have also experienced practical barriers in delivering quality end-of-life care and in establishing human connection with patients in palliative care [11].

In Canada, medical assistance in dying (MAiD) was initially legalized in 2016 under the federal Bill C-14 [12], and the legislation was subsequently amended in 2021 under Bill C-7 [13]. Competent adults are now eligible for MAiD if they have a serious and incurable medical condition, are in an advanced state of irreversible decline, and have intolerable physical or psychological suffering [14]. Procedural safeguards include a written request signed by one independent witness and assessments by two independent practitioners.

The COVID-19 pandemic has altered the delivery of MAiD services in Canada, with MAiD more often delivered in a home-based setting, fewer inter-facility transfers reducing MAiD access, and allowance of virtual witnessing and eligibility assessments [15–17]. Healthcare providers have reported challenges with MAiD access and logistics, perceptions of increased patient suffering, and moral distress related to enforcing institutional policies given the emotionality of the MAiD context [18–20]. However, to our knowledge, there have been no studies to date exploring patients and caregivers’ perspectives on the MAiD experience during the COVID-19 pandemic. The objective of this qualitative study was to understand how the COVID-19 pandemic has shaped the MAiD experience for patients and caregivers.

## Methods

### Study design and participants

Patients requesting MAiD and their family caregivers were recruited as part of a larger ongoing mixed-methods, multi-site study examining the MAiD experience. Recruitment for the larger study was conducted at two major academic health centres in Toronto, Canada, the University Health Network and the Sunnybrook Health Sciences Centre. Patients who requested MAiD in hospital and/or their caregivers who agreed to be approached for research purposes were identified by the clinical team to the research team. Eligibility criteria for participants were being 18 years of age or older, able to provide informed consent and complete study procedures in English, and for patients, having made a MAiD request.

For the present study, consecutive sampling of patients who requested MAiD and their family caregivers during the first year of the COVID-19 pandemic and their family caregivers was employed. Participants were invited to participate in a semi-structured interview about their experience with MAiD after a request by their family member. Six months following patient death, caregivers were interviewed to explore their bereavement experience. Informed consent was obtained from all participants.

### Data collection

Semi-structured interviews were conducted by the first author (ET) between April 2020 and May 2021. The interview guide included open-ended questions about the illness journey and healthcare received, MAiD decision-making, experience with the MAiD process, overall wellbeing, and support needs (see Additional File 1 for the full interview guide). Additional probes were included to inquire about the impact of COVID-19 throughout all aspects of experience unless they emerged organically. Interviews with patients, caregivers, and bereaved caregivers were analyzed together to triangulate and capture diverse perspectives at various timepoints of the impact of COVID on the MAiD experience.

The interviewer adopted a supportive, non-judgemental stance during interviews and debriefed weekly with the research team to discuss emerging insights and reflect on any pre-existing biases. All interviews were audio-recorded, transcribed verbatim, de-identified, and reviewed for accuracy prior to qualitative analysis.

### Data analysis

Transcripts were managed using qualitative software (NVivo10). Data were analyzed using reflexive thematic analysis to develop patterns of meaning across the data and generate reflective interpretations that may be useful to inform clinical practice and policy [21, 22]. The first author (ET) engaged in a process of data immersion and thoughtful reflection to develop initial codes, bringing together any initial observations or insights that arose during the interview phase. Codes were developed inductively to identify interesting and relevant aspects in the data. Initial codes were then organized and conceptualized into broader themes, aimed to create interpretive ‘stories’ about the data [22]. Throughout the analysis phase, ET met on a weekly basis with RN and SH to further refine the themes, review biases and any discrepancies, and promote ongoing reflexivity to reach fuller and more nuanced interpretations. Themes were then defined and conceptualized for broader clinical interpretations. Finally, the themes were further refined by co-authors, drawing upon their diverse perspectives and clinical experience in end-of-life care (e.g., palliative care (DS), bioethics (SB), psychiatry (SH, EIG, TT, GR, ML), psychology (RN)), while also acknowledging and questioning personal biases and persepctives as clinicians working in this context. Together, we sought to understand how participants experienced and made sense of MAiD in hospital during the COVID-19 pandemic.

### Ethics approval

The study was approved by the University Health Network Research Ethics Board (UHN REB #18–5227) and the Sunnybrook Health Sciences Centre Research Ethics Board (SHSC REB #106–2018).

## Results

### Participants

A total of 23 caregivers were interviewed. Of the 23 caregivers, 10 were interviewed at the time of the patient’s MAiD request only, 9 were interviewed during bereavement only, and 4 were interviewed both at the time of MAiD request and during bereavement, leading to a total of 35 interviews. In addition, seven patients were interviewed, of which six patients were interviewed on average 40 days prior to receiving MAiD in hospital. Participant characteristics for caregivers and patients are presented  in Tables 1 and 2, respectively. Interviews were conducted over the phone or in person and lasted on average 51 minutes.

**Table 1:** Caregiver participant characteristics (*n* = 23)

Characteristics	Mean (*SD)* or *n* (%); Range
Age	59 (11); 29–71
Gender (women)	14 (61%)
Ethnicity (White)	23 (100%)
Marital status	
Married/Common Law	18 (78%)
Separated/Divorced	4 (17%)
Single	1 (4%)
Religion	
Christian denomination	11 (48%)
None	7 (30%)
Jewish	4 (17%)
Other	1 (4%)
Highest level of education completed	
Undergraduate	9 (39%)
Post-graduate/Professional school	7 (30%)
College/Trade	4 (17%)
High school	3 (13%)
Combined family household income	
$30,000 to $59,999	4 (17%)
$60,000 to $99,999	4 (17%)
$100,000 to $199,999	6 (26%)
$200,000 +	6 (26%)
Do not wish to respond	3 (13%)
Living arrangement	
Living with spouse/partner	14 (61%)
Living with spouse/partner and children	5 (22%)
Living alone	3 (13%)
Living with children	1 (4%)
Relationship with patient	
Child	18 (78%)
Spouse/common-law partner	3 (13%)
Sibling	1 (4%)
Other family: child-in-law	1 (4%)
MAiD-eligible illness of patient	
Cancer	18 (78%)
Frailty/progressive weakness	3 (13%)
Extreme osteoporosis	1 (4%)
Severe airway obstruction/declined intervention	1 (4%)
MAiD status of patient	
Approved for MAiD	23 (100%)
Received MAiD	23 (100%)

**Table 2:** Patient participant characteristics (*n* = 7)

Characteristics	Mean (*SD)* or *n* (%); Range
Age	74 (12); 63–95
Gender (women)	5 (71%)
Ethnicity	
White	4 (57%)
Jewish	2 (29%)
East/Southeast Asian	1 (14%)
Marital status	
Single	2 (29%)
Widowed	2 (29%)
Married/Common Law	2 (29%)
Separated/Divorced	1 (14%)
Religion	
None	4 (57%)
Jewish	2 (29%)
Anglican	1 (14%)
Highest level of education completed	
High school or below	3 (43%)
College/Trade	2 (29%)
Undergraduate	1 (14%)
Post-graduate/Professional school	1 (14%)
Combined family household income	
< $14,999	1 (14%)
$15,000 to $29,999	1 (14%)
$30,000 to $59,999	1 (14%)
$60,000 to $99,999	2 (29%)
$200,000 +	2 (29%)
Living arrangement	
Living alone	3 (43%)
Living with spouse/partner	3 (43%)
Living with spouse/partner and children	1 (12%)
MAiD-eligible illness	
Cancer	6 (86%)
Hypertropic cardiomyopathy	1 (14%)
MAiD status	
Approved for MAiD	7 (100%)
Received MAiD	6 (86%)

### Findings

Our analysis generated 4 themes to capture the impact of the COVID-19 pandemic and its containment measures on the MAiD experience in hospital: accelerating the MAiD decision; compromising family understanding and coping; disrupting the MAiD day experience; and appreciating rule flexibility. Themes represented patterns of shared meaning across participants, beyond any differences of demographics, illness characteristics, or the severity of COVID-19 restrictions at the time of interview.

- (1) Accelerating the MAiD decision

The ever-evolving pandemic restrictions were described as causing “additional stress” and “needless suffering.” Participants described increasing feelings of isolation for patients and burden on families. These experiences were felt to have influenced the urgency of the MAiD decision, especially when the timeline and continued evolution of the public health threat were perceived to extend beyond the patient’s life expectancy:

COVID has been a big influence even on me choosing MAiD. Because of my timeline and [because] I really look at the world without any rose-colored glasses anymore […] I don't see me being alive long enough, so I just look at that, that I'm going to die in a world where I can't see anybody that I love. (Patient 225)

Participants described being a “victim of the COVID principles,” including appointment delays, availability and safety of home care, availability of specialized care, and limits on family members' physical presence. Compromised access to family and timely quality care was perceived to contribute to patient’s deterioration and their accelerated decision to pursue MAiD:

She had decided to do it anyway, but the virus only made it- and the restrictions around that- only made it more of an urgent decision. The fact is, she would be sitting there for ten days not being able to see anyone. And she was already declining and very uncomfortable and not eating and it was just intolerable to have to wait that long. (Caregiver 592-1)

- (2) Compromising family understanding and coping

Participants described how the pandemic impacted their family’s understanding of the reality of the patient’s illness progression and proximity to end of life. Caregivers were unable to see their loved ones as frequently as they would have wished or at all and realized that they may have “been living much longer in much more pain than we were aware.” Caregivers felt “surprised about how quick it was,” referring to their perception of the patient’s accelerated physical deterioration and MAiD process. Patients similarly faced challenges when conveying their decision around MAiD to loved ones who “didn’t see everyday changes,” making it “hard to accept”: “I discussed [MAiD] with everybody, and everybody was appalled, because nobody sees me because of COVID” (Patient 225).

The disconnected family understanding of the MAiD process was further complicated by “communication glitches.” There were often “notable” delays in relaying information from the healthcare team to families, who were “separated by circumstances” and restricted from hospital visits. For those permitted to visit in a limited and staggered capacity, it was challenging to achieve mutual understanding across family members. One participant shared, “We couldn’t all three – my sister, my mother and I – talk to her at the same time. So we couldn’t visit her at the same time to try to get on the same page about everything” (Caregiver 604–1).

The impact of hospital visitor restrictions on communication and interaction was described as “very awful, not knowing,” “a gigantic strain on patients and families,” “absurd,” and “inhuman.” Caregivers allowed sole visitation permission felt the sense of responsibility was “too big a cross for one person to bear.” These collective experiences caused immense emotional distress:

It’s absolutely been terrible to only have two hours a day with my mother. That’s absolutely absurd. I know under normal circumstances we would be allowed there the whole day, right? Or even stay the night like if that was even possible. So yeah, it’s made a huge impact on everything. So my time is already limited, and now somebody is making it more limited. (Caregiver 616-1)

- (3) Disrupting the MAiD day experience

Hospital visitor restrictions on the day of MAiD were “difficult to accept.” The reality of not being able to be physically present was incredibly distressing:

I think the doctor had asked if we wanted to Skype and honestly, I remember when she asked that and I thought I was going to throw up. I literally have that visceral memory thinking that– I couldn't [starts crying] imagine seeing my father for the last time on a computer screen. (Caregiver 618-1)

When one visitor was allowed to be present, participants described the familial distress around the “terrible decision” to choose which family member would be present:

We had to decide who would be there at the last moment. My little sister standing outside the hospital in the cold, on a phone–thank God Dr. X allowed her to use FaceTime on her phone, so having my sister’s face in a Ziploc bag on an iPad at my dad's knee was just- […] I don't know how I ever get over her face on that iPad when she couldn't be in the room at that time. (Caregiver 612-1)

Another caregiver described:

[My mother’s sister] couldn’t even be in the room while she died, she had to wait downstairs in the lobby. Like that’s a special kind of sadness, like it is special and reserved for only a very few people in this world where they’re denied entry to see someone on their final day. (Caregiver 633-1)

Personal protective equipment (PPE) protocols further compromised the MAiD day experience. One patient shared:

What I wanted to do was have a pizza party […] And I can be the only one that eats because of COVID, you can't have a bunch of people with their masks off eating. So I will be the only one that eats at my pizza party. (Patient 223)

Participants described the emotional impact of the mask requirements on such an intimate, finite, and relational experience as death: “Obviously everybody has to be masked […] but my dad was such a jokester. The fact that he couldn't see people laughing at his last joke was very painful to me” (Caregiver 612–1).

- (4) Appreciating rule flexibility

Participants acknowledged the burden of “overworked” staff. They expressed their appreciation for those who went “out of their way to accommodate” their needs during such challenging circumstances and against evolving hospital restrictions, which often felt “arbitrary”:

There is some serious shortcomings in the arbitrary nature of these rules, especially the people who are at their end of life. I think that one of the greatest differences that can be looked at, would be just to allow people to see who they want to see before they die […] Coronavirus is dangerous, it’s got to be managed, I get that. But this isn't the way! To deny patients of their loved ones is not the way. It’s inhuman (Caregiver 633-1).

Another caregiver recalled, “They were doing everything they could to work around all of their parameters that I’m sure must have been very frustrating for them” (Caregiver 613–1). Participants reflected on the profound impact of small gestures and granting “bent” rules by healthcare providers on the end-of-life experience, especially when nearing MAiD day itself:

They told me kind of last minute. I was there on Thursday night. She had [MAiD] on a Friday. And [Doctor] called me, I guess she was just on her way out, she said, ‘just so you know, you're more than welcome to stay the whole night.’ And I said, ‘I would love to do that,’ and I did. I slept in my same clothes. I stayed the whole night there. My wife brought me a delivery of some Chinese food. Mom wanted some Chinese food. She brought my guitar because Mom wanted me to play on the day that she died and I got everything delivered and we stayed in. It was just like any other night, except for the obvious. And it was really helpful, really helpful to her (Caregiver 633-1).

On the day of MAiD, these acts of compassion were most profound. Participants felt “extremely grateful” when approval was granted for multiple people to attend and rules were applied flexibly:

The accommodation that the people on the palliative floor have gone out of their way to be able to get the proper approvals and then let our granddaughter go in as opposed to just myself, I think it’s just excellent […] To us, that would be climbing Mount Everest, but they find a way of accommodating you, and I think that experience is absolutely overwhelming (Caregiver 623-1).

For caregivers who were granted exceptions to rules, they expressed their gratitude towards the healthcare providers in helping them restore elements of the ideal dying MAiD experience. One caregiver reflected:

I wanted to kiss her. I wanted to kiss her hand. I wanted her hand against my head, my face. I was able to touch her before, but I had had a mask on. But I asked the doctor, ‘May I take my mask off,’ and she said yes […] That was very important for me during the procedure. I didn't have it off until that last half hour […] Her vision was impaired at the end. That morning she had told me, ‘I can't see you.’ That disturbed her, so I wanted her to feel me and I wanted her to feel my face (Caregiver 604-1).

## Discussion

To the best of our knowledge, our study is the first qualitative report of the impact of COVID-19 pandemic on the experience of MAiD and is aligned with calls for rapid qualitative research to complement clinical research and epidemiological data during the pandemic [23, 24]. Our analyses identified that the pandemic and its containment measures were associated with accelerating the MAiD decision, compromising family understanding and coping, disrupting the MAiD day experience, while also appreciating the flexible application of rules and policies to allow for an optimal experience.

Our findings highlight the tensions between COVID-19 restrictions and individual control over the circumstances of dying, and the resulting impact on patient and family suffering. Previous research in Canada and other jurisdictions with legalized assisted dying have consistently shown that MAiD requests are primarily driven by concerns about loss of autonomy and dignity, loss of ability to engage in meaningful activities, and burden on family and friends [16, 25, 26]. Our results further highlight the extent to which MAiD is not only a response to suffering but also the need to orchestrate a desired death experience. Individual needs and plans were compromised in many ways throughout the pandemic.

In our study, patients’ accelerated MAiD decision-making was related to suffering in isolation and health system constraints, congruent with MAiD providers’ perceptions of increased suffering and case urgency and reduced available services [18, 27]. Our finding that COVID-19 circumstances interfered with families’ understanding and ability to cope highlights the need for institutions to prioritize family inclusion and presence in order to reduce isolation and distress for both patients and families. Indeed, caregivers who are more actively involved in the MAiD process have been shown to perceive and to cope with the process more favourably [28, 29]. The compromised nature of MAiD day, due to visitor restrictions and PPE requirements, may lead to greater caregiver distress, as caregivers often experience an obligation to orchestrate the ideal dying experience for their loved one [29]. Navigation through pandemic restrictions to organize MAiD day has similarly been identified as a stressor for MAiD providers [18].

COVID-19-related restrictions compounded participants’ suffering, which was often mitigated through compassionate interactions with the healthcare team. Participants expressed their gratitude towards flexible application of rules and policies. This echoes the moral tension identified by healthcare providers between respecting hospital policies and meeting their perceived standards of quality care [11]. Similarly, MAiD providers have reported instances where they did not follow or insist on public health rules during MAiD provision [19]. They emphasized the “exceptionality” of the MAiD context during COVID-19 and the need for “compassionate exceptions.” Due to these tensions, providers may experience heightened cognitive dissonance and moral injury during and beyond COVID-19 [30].

Our findings emphasize the social and relational influences on the quality of the MAiD experience [31], despite the focus within MAiD legislation and implementation discussions on patient autonomy. The profound impact of restricted physical presence and connection on the wellbeing of patients and families in our study raises important ethical questions about the importance of humane, family-centered care at the end of life. A patient’s wish for meaningful and personalized control over their death must also be understood in the context of their family system, acknowledging the increased reliance on family at end of life and recognizing the support needs of loved ones. Healthcare institutions and systems must reflect on the ethical values underlying MAiD and end-of-life care policies both amid the COVID-19 pandemic and beyond. We can draw on strategies recommended for effective, humane hospital policies that acknowledge the value of social support during illness and death in the COVID-19 context [5, 32, 33], as well as lessons learned from previous outbreaks such as SARS 2003 [34, 35]. These considerations may help inform not only preparation and risk mitigation strategies for future similar public health crises but also help to clarify the priorities that shape and guide health care more broadly.

## Limitations

Our study is limited by response bias; it is possible that individuals with the most complicated and distressing MAiD experiences were less able or willing to participate. There was also insufficient representation from those who received MAiD in the community and in private residences, which have increased significantly during COVID-19 in 2020 compared to in-hospital provision [16]. In relation to the present study findings, further exploration to understand whether the pandemic has influenced individuals’ decisions to pursue MAiD and die at home is recommended. The sample is also predominantly White, relatively affluent, and most patients had a cancer diagnosis. Although these distributions are aligned with MAiD request data in Ontario [36, 37], further understanding of the experiences of individuals of different ethnic and racial identities and socioeconomic and disease backgrounds is needed.

## Conclusions

This study provides valuable in-depth insight into how the COVID-19 pandemic has transformed the MAiD and end-of-life landscape in Canada. Beyond prioritizing autonomy and minimizing suffering, our results highlight the significance of the MAiD option for patients and families, allowing orchestration of an ideal death experience, although this was compromised in several ways throughout the pandemic. Healthcare settings that value and prioritize family involvement in MAiD and end-of-life care, and that support clinicians in attempts to balance this in the face of public health and infection control priorities, may minimize distress and support wellbeing of patients, families, and providers. This study may inform strategies and risk–benefit analyses to optimize the MAiD experience through hospital policies and resource allocation during the pandemic and beyond.

## Supplementary Information

**Additional file 1. **

## Data Availability

The datasets generated and/or analysed during the current study are not publicly available due the protection of participants’ identities but are available from the corresponding author on reasonable request and following application to the University Health Network Research Ethics Board and Sunnybrook Health Sciences Centre Research Ethics Board.

## Abbreviations

COVID-19: Coronavirus disease 2019
MAiD: Medical assistance in dying
PPE: Personal protective equipment
