# Supplementary material for: The impact of COVID-19 on the experiences of patients and their family caregivers with medical assistance in dying in hospital
Source: BMC Palliat Care. 2023 Jun 13;22:70. doi: 10.1186/s12904-023-01191-8 (PMC10261826; doi:10.1186/s12904-023-01191-8)
Supplement: Supplementary file 1 — Additional file 1. [file 12904_2023_1191_MOESM1_ESM.docx]

**Interview Guide**

**Interview Protocol for Patients**

**Introduction**

This interview is being conducted to understand your current experience with the process of Medical Assistance in Dying (MAID). My goal is to understand as deeply as I can your own experience. Some of these questions are very personal and may be difficult to talk about, so please let me know if you become uncomfortable at any time. If at any time you need a break or need to stop, or if there are any questions you don’t want to answer, please let me know.

**Opening Questions**

- Can you tell me a bit about yourself? And can you tell me a bit about your illness?
- [If participant finds it difficult to answer, probe: Can you tell me how are you doing in terms of your overall well-being? Can you tell me how are you doing in terms of your physical well-being? Can you tell me the ways in which your illness has affected your life? What is most important to you in your life right now?]
- Can you tell me about your experience with MAID up to this point?

**Interview Questions regarding MAID** to be integrated in the conversation when appropriate

1) Initial decision

- Can you tell me how you made your decisions around MAID?
- When was it that you began to think about the idea of MAID?
- Can you tell me your reasons for wanting to receive MAID?
- While you were thinking about asking for MAID, did any reasons not to ask for MAID cross your mind? Can you describe them to me?
- Do you recall any thoughts (if any) that you had about MAID before considering MAID as an option yourself?
- Do you know anyone who requested MAID? Received MAID? What, if anything, did you learn from that person’s experience?
- [if unclear] How long was it between the time you first started contemplating the idea of MAID and between requesting it for yourself?
- One of the eligibility criteria for MAID is enduring physical and psychological suffering that is intolerable, can you tell me what that means to you? And since when you feel you have experienced this kind of suffering?
  - How would you describe suffering?

2) Sharing the decision with others

- Have you discussed the idea of MAID with anyone else? Who did you discuss it with? What was it like to talk to others about MAID? What did they have to say? What was their initial reaction? Have their views and feelings affected your decision in any way? How so? [probe about spouse/caregiver, family members, friends, and health care providers, including palliative care providers, if applicable]
- What surprises, if any, did you encounter when you informed others of your decision to consider and/or proceed with MAID?
- What positive outcomes, if any, have resulted from sharing your decision to consider and/or proceed with MAID with others?
- Were there any barriers to discussing the idea of MAID with others? Have you avoided discussing the idea of MAID with certain people? Why do you feel you cannot talk with these people?
- Do you wish there were people you could discuss this experience with but can’t? If yes: Who would they be?

3) MAID Resources

- Do you think you have enough information and understanding about MAID? How did you receive this information? Is there anything you have wished you knew earlier about MAID? What, if anything, is unclear or confusing for you about MAID?
- Which resources were helpful for you while going through the MAID process? Do you feel you needed any additional resources while going through the MAID process? If yes: can you elaborate?

4) Quality of Care

- How, if at all, did the approach to or quality of your care change when your team learned of your request for MAID?
- What is your perspective on the health care you have received up until now? What was good? What was not good? Has your care impacted your decisions around MAID in any way? How so?
- One perspective on MAiD is that it can be seen as a failure of the healthcare system. What are your thoughts on this point of view?
- Have you at any point received any psychosocial support (e.g., met with a social worker, a psychologist, or a psychiatrist) and/or palliative care services? Were they ever offered to you or to your family? [if yes] When was that? What was your experience with these services so far? Have these services impacted your decisions around MAID in any way? How so?

5) The MAID process

- Can you tell me what happened when you requested MAID? Who did you raise this issue with (e.g., oncologist, nurse)? What was the conversation like?
- [If participant completed a MAID assessment] What was the MAID assessment like for you? What are the next steps?
- [If participant was approved for MAID] What are the next steps? When would you like MAID to take place? What do you imagine it would be like? Do you have any concerns or worries about how it will happen or when? Do you have any moments of fear or doubt? What do you feel that you need from your family or health care providers at this stage?
- [If participant withdrew his/her request prior to the MAID assessment] Can you tell me about your decision not to go through with MAID? What are the next steps for you? What do you feel that you need from your family or health care providers at this stage?
- Thinking about your experience with the MAID process so far, what do you think about the process overall? is there anything that can be done differently to make it easier for you and/or your family? Is there anything that surprised you about this process? Anything that you did not expect? Did your initial perception of MAID change now that you went through this process yourself?
- Having gone through the MAID process, what is your take on the possibility of allowing patients to include MAID as an advance care directive? What about to consider MAID for mature minors, or those younger than 18 years of age?

**Before completion**

Summarize and check understanding of themes raised in the interview.

Probe: Is there anything else that you would like to add? Is there any important issue in your life or about MAID that we haven't talked about and that you would like to share with me?

Probe: How have you felt about taking part in this interview? Is there anything you would like to ask me about the interview?

**Interview Protocol for Caregivers**

**Introduction**

This interview is being conducted to understand your current experience with the process of Medical Assistance in Dying (MAID) initiated by your loved one. My goal is to understand as deeply as I can your own experience. Some of these questions are very personal and may be difficult to talk about, so please let me know if you become uncomfortable at any time. If at any time you need a break or need to stop, or if there are any questions you don’t want to answer, please let me know. We can always reschedule the interview if you feel unable to fully participate today.

**Opening Questions**

- Can you tell me a bit about yourself? Can you tell me a little bit about your loved one? Can you tell me a bit about your loved one’s illness?
  - [If participant finds it difficult to answer, probe: Can you tell me how your loved one is doing in terms of his/her overall well-being? How do you spend time together?
- Can you tell me the ways in which his/her illness has affected your life? What kind of practical and/or emotional issues came up for you during your loved one’s illness?]
- Can you tell me about the MAID process up to this point from your perspective?

**Interview Questions regarding MAID** to be integrated in the conversation when appropriate

1) Initial decision

- How do you think your loved one arrived at the idea of MAID?
- What do you think about your loved one’s decision? Can you tell me your reasons for wanting or not wanting him/her to receive MAID? What was the decision making process like? How involved do you feel in making this decision?

2) Conversations about MAID

- Has your loved one discussed the idea of MAID with you? When? What did s/he say? What was your initial reaction? Did your reaction change over time? What was it like for you to have this discussion?
- Have you discussed your loved one’s interest in MAID with anyone else? Who did you discuss it with? What was it like to talk to others about this? What did they have to say? What was their initial reaction? Have their views and feelings affected your view in any way? How so? [probe about family members, friends, and health care providers, including palliative care providers, if applicable]
- Were there any barriers to discussing the idea of MAID with others? Have you avoided discussing the idea of MAID with certain people? Why do you feel you cannot talk with these people? [probe about family members, friends, and health care providers, including palliative care providers, if applicable]
- At any point in the process, did you try to dissuade your loved one? Why? How did you make your position known?
- Do you wish there were people you could discuss this experience with? If yes: Who would they be?
- What are your greatest worries or concerns related to your loved one’s consideration of MAID?

3) MAID Resources

- Do you think you have enough information and understanding about MAID? How did you receive this information? Is there anything you have wished you knew earlier about MAID? Is there anything that is unclear or confusing for you about MAID?
- Which resources were helpful for you while your loved one is going through the MAID process? Do you feel you needed any additional resources? If yes: can you elaborate?

4) Quality of Care

- What is your perspective on the health care your loved one has received up until now? What was good? What was not good? Do you think his/her care impacted his/her decisions around MAID in any way? How so? Do you think it impacted your views about MAID in any way? How so?
- Have you at any point received any psychosocial support (e.g., met with a social worker, a psychologist, or a psychiatrist)? Were they ever offered to you or to your loved one? [if yes] When was that? What was your experience with these services so far? Have these services impacted your experience with MAID in any way? How so?
- What, if any, were your perceived changes in the approach to or quality of care your loved one received after making the decision to proceed with MAID?

5) The MAID process

- Can you tell me what happened when your loved one requested MAID? How involved were you in this process?
- [If patient completed a MAID assessment] What was the MAID assessment process like for you? Have you been part of it? In what ways? What are the next steps?
  - What was your impression of the clinicians who provided assessment for eligibility for MAID with your loved one?
- [If patient was approved for MAID] What are the next steps now that your loved one is approved for MAID? What do you imagine MAID would be like? Do you have any concerns or worries about how it will happen or when?
- [If participant withdrew his/her request prior to the MAID assessment] Can you tell me about your loved one’s decision not to go through with MAID? What are the next steps?
- Thinking about your experience with MAID so far, what do you think about the process overall? is there anything that can be done differently to make it easier for you and/or your loved one? Is there anything that surprised you about this process? Anything that you did not expect?
- What is your take on the possibility of allowing patients to include MAID as an advance care directive?

**Before completion**

Summarize and check understanding of themes raised in the interview.

Probe: Is there anything else that you would like to add? Is there any important issue in your life or about MAID that we haven't talked about and that you would like to share with me?

Probe: How have you felt about taking part in this interview? Is there anything you would like to ask me about the interview?

**Interview Protocol for Bereaved Primary Caregivers**

**Introduction**

This interview is being conducted to understand your bereavement experience. My goal is to understand as deeply as I can your own experience. Some of these questions are very personal and may be difficult to talk about, so please let me know if you become uncomfortable at any time. If at any time you need a break or need to stop, or if there are any questions you don’t want to answer, please let me know. We can always reschedule the interview if you feel unable to fully participate today.

**Opening Questions**

1) Can you tell me what life is like for you right now?

2) Can you tell me about the final days and death of your loved one?

**Interview Questions regarding MAID and Grief** to be integrated in the conversation when appropriate

- Were you present for the death of your loved one? Could you describe his/her death? If you weren’t present, could you describe what you were told about the death? Do you feel you were able to say goodbye?
- What is your perspective on the health care your loved one received around the time of their death, including any involvement with psychosocial support (e.g., social work, psychology, psychiatry) and/or palliative care services? What was good? What was not good?
- [if patient received MAID] How was the MAID process from your perspective at the end? What was the setting like at the time of death? Who was present? What were the interactions with the health care team like at the time of death? Were there any additional professionals present (e.g., music therapist)? Was there anything that could have been done differently to make it easier for you and/or your loved one? Is there anything that surprised you about it? Anything that you did not expect?
- Can you recall what your feelings were when your loved one passed away? Are you still dealing with the same feelings now as you did at the time of death? What is it like for you now?
- How do you think the fact that [the patient received/did not receive MAID] affected his/her death? Your experience of his/her death? And your grief after his/her death?
- What deaths of other loved ones have you have experienced? How is your current experience of grief similar to your grief from these other deaths you have experienced? How is it different?
- How did other people react to your loved one’s death? [if patient received MAID] How did you describe the death to others? How did people react to the fact that your loved one received MAID?
- What was offered to you in terms of grief support? Did you use any grief resources offered? If yes: What and when? What was it like? What was helpful/unhelpful about these resources? If not: Why not? What would you have liked to be offered? And when?
- Thinking about your experience of supporting your loved one, what is your take on the possibility of allowing patients to include MAID as an advance care directive?
- What was your impression of the clinician who provided MAID to your loved one?

**Before completion**

Summarize and check understanding of themes raised in the interview.

Probe: Is there anything else that you would like to add? Is there any important issue in your life or about your grief that we haven't talked about and that you would like to share with me?

Probe: How have you felt about taking part in this interview? Is there anything you would like to ask me about the interview?
